# Production Strategies of TiN_x_ Coatings via Reactive High Power Impulse Magnetron Sputtering for Selective H_2_ Separation

**DOI:** 10.3390/membranes11050360

**Published:** 2021-05-15

**Authors:** Cecilia Mortalò, Silvia Maria Deambrosis, Francesco Montagner, Valentina Zin, Monica Fabrizio, Luca Pasquali, Raffaella Capelli, Monica Montecchi, Enrico Miorin

**Affiliations:** 1 National Research Council of Italy—CNR, Institute of Condensed Matter Chemistry and Technologies for Energy—ICMATE, Corso Stati Uniti 4, 35127 Padova, Italy; cecilia.mortalo@cnr.it (C.M.); francesco.montagner@cnr.it (F.M.); valentina.zin@cnr.it (V.Z.); monica.fabrizio@cnr.it (M.F.); enrico.miorin@cnr.it (E.M.); 2 National Research Council of Italy—CNR, Engineering ICT and Technologies for Energy and Transportation Department, Piazzale A, Moro 7, 00185 Roma, Italy; 3 Dipartimento di Ingegneria E. Ferrari, Università di Modena e Reggio Emilia, via Vivarelli 10, 41125 Modena, Italy; luca.pasquali@unimore.it (L.P.); raffaella.capelli@unimore.it (R.C.); monica.montecchi@unimore.it (M.M.); 4 IOM-CNR, s.s. 14, Km. 163.5 in AREA Science Park, Basovizza, 34149 Trieste, Italy; 5 Department of Physics, University of Johannesburg, P.O. Box 524, Auckland Park 2006, South Africa

**Keywords:** TiN_x_ film membranes, high power impulse magnetron sputtering, chemical robustness under h_2_, porous ceramic substrates

## Abstract

This scientific work aims to optimize the preparation of titanium nitride coatings for selective H_2_ separation using the Reactive High Power Impulse Magnetron Sputtering technology (RHiPIMS). Currently, nitride-based thin films are considered promising membranes for hydrogen. The first series of TiN_x_/Si test samples were developed while changing the reactive gas percentage (N_2_%) during the process. Obtained coatings were extensively characterized in terms of morphology, composition, and microstructure. A 500 nm thick, dense TiN_x_ coating was then deposited on a porous alumina substrate and widely investigated. Moreover, the as-prepared TiN_x_ films were heat-treated in an atmosphere containing hydrogen in order to prove their chemical and structural stability; which revealed to be promising. This study highlighted how the RHiPIMS method permits fine control of the grown layer’s stoichiometry and microstructure. Moreover, it pointed out the need for a protective layer to prevent surface oxidation of the nitride membrane by air and the necessity to deepen the study of TiN_x_/alumina interface in order to improve film/substrate adhesion.

## 1. Introduction

The sustainable extraction of pure H_2_ from a mixture (reformate from hydrocarbons and WGSR or biomass processing) can be considered an enabling technology for using H_2_ as an energy carrier or chemical reagent. In fact, this process is a critical step in the reliable production of cheap hydrogen. Although H_2_ is a precious energy carrier and an irreplaceable reagent in the synthesis of numerous chemicals, it does not exist in nature as a free gas. An abundant supply of H_2_ is derived from fossil fuel sources (>90% of global H_2_ production) and by gasification of coal, but also by steam reforming of upgraded biogas or by syngas. Then, H_2_ must be separated from the resulting reformer and/or water-gas shift reaction mixtures, which contain other products such as CO, CO_2_, N_2_, H_2_S, NH_3_, and H_2_O. The methods industrially exploited for hydrogen purification are pressure swing adsorption (PSA) [1] and cryogenic distillation. Both are effective and reliable but require severe pressure and temperature conditions being also highly energy-consuming. A possible alternative is represented by membrane technologies, in particular, those that use dense membranes, where the separation of hydrogen occurs selectively thanks to the chemical-physical properties of the active material. The studies conducted so far have shown that membrane technologies could have significant advantages, including low energy consumption, the ability to perform continuous hydrogen separation, and the possibility of an industrial scale-up [2].

Currently, H_2_ membranes at an advanced stage of engineering are based on palladium and its alloys [3], which have proved to be the most functional in terms of efficiency and industrial integration. Although a range of H_2_-selective materials has been developed, none of these has been able to rival Pd alloys in terms of efficiency.

However, the elevated cost of the metal [4] requires a strategy change. Potential alternatives to Pd and group IV and V metals are mixed protonic-electronic conductors (MPEC) with ambipolar diffusion of H^+^ and e^−^. In particular, perovskite-based mixed protonic–electronic conducting membranes are deeply investigated [5,6,7,8,9]. These materials incorporate hydrogen in the lattice as proton defects that are associated to the oxide anions. In general, the site-to-site proton conduction mechanism in these oxides requires relatively high activation energy; therefore, MPECs are normally used at high temperatures (typically T > 700 °C).

On the other hand, theoretical and experimental studies conducted on semiconductor nitrides and oxides have shown that in some cases, hydrogen acts as a dopant and contributes to increasing the electrical conductivity of the semiconductor [10,11]. This is the opposite of what is normally expected; more specifically that H could assume donor character in p-type (H^+^) semiconductors and acceptor in n-type (H^−^) semiconductors, thus counteracting the prevailing conductivity. For these nitrides, by applying an appropriate pressure difference to the two sides of a thin membrane, it was observed that hydrogen selectively permeated the material and diffused following the direction of the pressure gradient.

This peculiar behavior was observed and quantitatively described in HfN [12], used as a component of multilayer membranes for the isotope separation of hydrogen, and in TiN [13,14,15], and confirmed by DFT (Density Functional Theory) calculations conducted on TiN_x_. The most recent studies verified H permeability experimentally; it was attributed to the formation of Ti-H terminal groups on the surface of crystallites in nanocrystalline matrices and the subsequent diffusion of H^-^ through the boundary-grain interphase. The exchange mechanism between Ti-H end groups has a low activation energy (5 kJ·mol^−1^). The permeation rate measured through the TiN_0.7_ films was found to be approximately 1 × 10^−6^ mol·cm^−2^·s^−1^ at 25 °C, i.e., higher than that observed in Pd membranes at the same temperature [14]. TiN-based materials are well-known and used for engineering applications thanks to the combination of high hardness, corrosion resistance, chemical inertness, and a low friction coefficient [16,17]. The constituent elements are readily available and economically sustainable, and these studies represent the starting point for investigating the possible use of TiN_x_ coatings as alternative membranes for the selective separation of hydrogen at intermediate temperatures (25–500 °C) [14].

As already mentioned, the transport of hydrogen through the TiN_x_ membrane occurs through a bond exchange mechanism between the Ti-H tails attached to neighboring crystalline grains of the film. According to this premise, this study has been conceived to optimize a Physical Vapor Deposition (PVD) method for the preparation of TiN_x_ coatings that must fulfill the following conditions: (i) first, to achieve fine control of the composition since the H_2_ absorption could be related to stoichiometry; (ii) secondly, to pursue a nanocrystalline TiN_x_ structure because a high number of grains favors the presence of unbound Ti at the grain boundaries, available for the formation of Ti-H bonds; (iii) finally, to deposit dense and homogeneous TiN_x_ films in view of potential membrane engineering.

Therefore, based on these considerations, reactive HiPIMS technology was used for depositing TiN_x_ coatings on suitable dense (silicon) and porous (alumina) substrates. Magnetron sputtering (MS) is a PVD method that permits the depositing of high-quality layers and is broadly applied in the industry (semiconductors and electronics, tools and decorative films, glass windows, optical coatings, photovoltaics, etc.). MS coatings typically show a columnar microstructure [18]. Size, morphology, and relative orientation of crystallites are governed by growth chemistry [18] and the effect of surface and bulk diffusion processes (controlled by, for example, the substrate temperature during deposition). With respect to traditional MS techniques, HiPIMS plasma conditions greatly improve the flux of low energy ions that irradiate the growing film, enhancing adatom mobility. This energetic bombardment affects nucleation density and crystallographic orientation and allows the synthetizing of smoother and denser coatings to be deposited even on insulating and complex-shaped substrates [19]. Reactive HiPIMS is used to grow compound films and can be prepared from a metal target by the addition of a reactive gas (i.e., for instance, N_2_) to a noble working gas (generally Ar) [20].

In this pilot research, TiN_x_ coatings were deposited via RHiPIMS on silicon substrates using a pure titanium target and a mixture of Ar and nitrogen. Sputtering parameters, such as substrate temperature and N_2_/Ar ratio, were changed to tune composition and microstructure. Then, appropriate working conditions were set up to grow the coating on porous alumina substrates. Alumina was chosen because it should be mechanically and chemically stable under the operating conditions of the membranes (up to 500 °C). Alumina substrates also have the advantage of preventing interdiffusion phenomena, typical of steel substrates, that could drastically reduce hydrogen permeation [3]. Furthermore, alumina is a relatively cheap material, and in view of an industrial scale up of these systems, the cost of porous substrates can significantly affect the final price of the device. Finally, to assess their chemical stability and robustness, TiN_x_ films were subjected to high temperatures (i.e., 500 °C) under an H_2_-containing atmosphere.

## 2. Materials and Methods

TiN_x_ coatings were deposited via reactive HiPIMS starting from a ~10 cm diameter of pure Ti target (99.9%). A spherical vacuum chamber was used in combination with the TRUMPF-Hüttinger power supply (Tru Plasma High Pulse 4002) and the bias unit model 3018 HBP (18 kW). Firstly, 0.6 mm thick silicon wafers (100) were used as test substrates to start fine-tuning the deposition process. Si does not require any surface preparations before deposition, except for a short ultrasonic washing, rinsing in ethanol, and rapid drying. Then, one test sample was successfully grown on a porous alumina substrate. Commercial substrates were supplied by Cobra Technologies BV (Rijssen, The Netherlands) [12,14]. The substrates are discs with a 25 mm diameter, a 2 mm thickness, consisting of a support of α-Al_2_O_3_ and having an 80 nm controlled porosity; they are polished (Ra < 0.1 µm) and coated on one side with a layer of γ-Al_2_O_3_ that has a nanometric porosity in the range of 3 to 5 nm.

The total working pressure was arbitrary set at 5 × 10^−3^ mbar (Ar 99.999% plus N_2_ 99.998% purity), the substrate temperature was kept at 300 °C (140 °C for sample TiN1), the selected average power was 500 W, HiPIMS frequency was 500 Hz, and pulse length was equal to 25 s. The applied substrate bias was 100 V (DC), target-sample holder distance was fixed at 80 mm, while deposition duration was 120 min. As reported in Table 1, the amount of nitrogen (reactive gas) within the process mixture has been changed from 1 to 3 sccm in order to vary the properties of the films.

To evaluate deposition rates, the thickness of the coatings was measured using a ball crater micro-abrasion method (Calotest Anton Paar). The surface morphologies of deposited films were investigated by FE-SEM (Sigma Zeiss) and the chemical composition of TiN_x_ coatings was measured by energy-dispersive electron probe microanalyses (EDS, Oxford X-Max). Moreover, the crystal structure was evaluated by X-ray diffraction (XRD, Philips PW 3710) operating both in grazing angle (angle of incidence ω of 2°) and Bragg-Brentano geometry and equipped with a Cu-Kα source (40 kV, 30 mA). XRD spectra were collected, selecting a 2θ scanning range from 30 to 85°, and were first evaluated with the Match! 2.2.1 Software to identify the crystalline phases from the position of detected peaks. Then profile fits were elaborated using Maud 2.8 software [21,22], through the iterations of the Rietveld method, applied to peaks broadening and positions [23].

Furthermore, electron photoemission spectroscopy measurements were carried out using an ultra-high vacuum system (base pressure 2 × 10^−8^ Pa) equipped with a hemispherical electron analyzer (VG Microtech model CLAM2) and double anode X-ray source (Al and Mg) (VG Microtech model VG XR3). In the present analysis, we used non-monochromatized Mg K_alfa_ photons (1253.6 eV), and an overall resolution of 0.5 eV (photon and analyzer) was selected.

To investigate the chemical stability under operational conditions typical for hydrogen separation membranes, four representative samples were treated at 500 °C for 20 h under an H_2_-containing atmosphere. Thermal treatments were conducted using a Nabertherm N 11/HR tubular oven. After degassing the samples in a vacuum, the treatment was carried out in Ar/3%H_2_ (2 L/min) with a heating rate of 200 °C/h. Samples were kept at 500 °C for 20 h, and, finally, the system was cooled down to RT in 2.5 h.

## 3. Results and Discussion

### 3.1. As Deposited Coatings

Table 1 shows the samples deposited in reactive mode as the partial pressure of nitrogen varies from 2.8 × 10^−3^ Pa (1 sccm) to 9.5 × 10^−3^ Pa (3 sccm). Samples TiN1 and TiN2 are grown under the same HiPIMS conditions, except for the substrate temperature, which is equal to 140 and 300 °C, respectively. TiN4A was deposited on a commercial porous alumina substrate, setting the same working parameters used for the synthesis of the TiN4 film.

**Figure 1:**
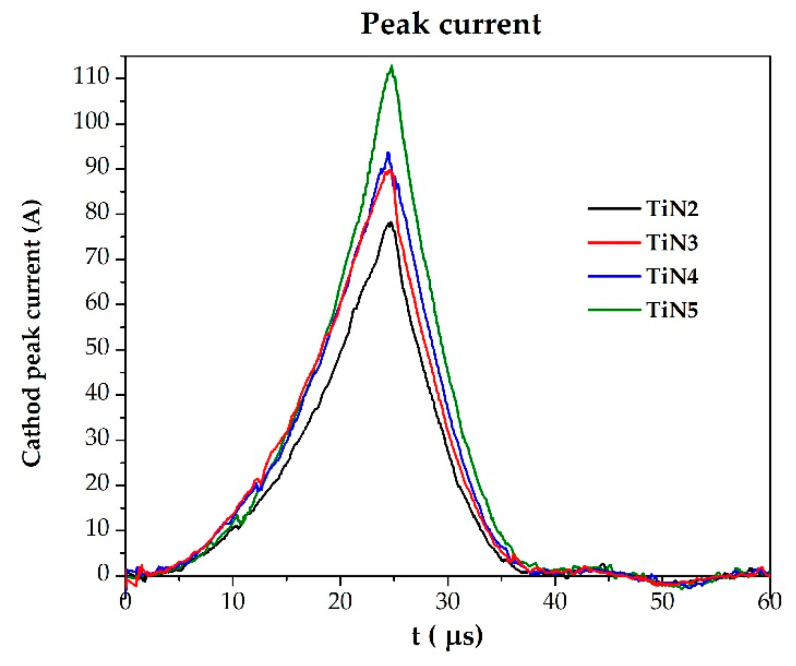
HiPIMS peak current variation (pulse time = 25 μs) while changing the nitrogen flux during the deposition process. The example cases of TiN2 (1 sccm N_2_), TiN3 (1.2 sccm), TiN4 (1.5 sccm N_2_), and TiN5 (2 sccm N_2_) are shown.

Using HiPIMS, high power discharge pulses are applied to the target, and large discharge currents are generated. The presence of the reactive gas leads not only to the desired compound formation at the substrate but also to its simultaneous chemisorption at the target that is covered with a compound layer (i.e., target poisoning). Increasing the poisoning extent (i.e., growing the nitrogen quantity from 1 to 3 sccm) determines the evolution of the deposition process. It passes from *metal mode* (i.e., low argon repeated use, “recycling” during the sputtering process) to *compound mode* (i.e., high argon recycling). As a consequence, the corresponding discharge currents stabilize at different amplitudes, as shown in Figure 1, where the peak current curves for samples TiN2, TiN3, TiN4, and TiN5 are reported. This results in the depositing nitride coatings having different properties, as widely described below.

From Table 1, a decrease in the deposition rate as the nitrogen flow increases is observed: this behavior relates to target poisoning. As widely reported in the literature, the progressive increase in Ti target poisoning (i.e., >process N_2_) leads to a net decrease in the sputtering yield because Ti-N bond strength is greater than Ti-Ti bond strength. As a consequence, the deposition rate reduces accordingly [20].

When comparing TiN1 e TiN2, despite the constant working parameters, the film thickness slightly decreases when the temperature increases from 140 °C to 300 °C. Indeed, as reported in [24], the films should become denser thanks to the higher particle mobility.

Table 2 shows the chemical composition values measured by SEM-EDS for as-deposited TiN_x_ coatings and after the exposure to the Ar/H_2_ mix at 500 °C. In regards to as-deposited TiN_x_, as expected, while the nitrogen/argon ratio increases, there is a rise in the amount of elemental nitrogen in the coating. At constant deposition temperature, x moves from 0.66 (sub stoichiometric composition), when the N_2_ partial pressure is equal to 2.8 × 10^−3^ Pa mbar, to 1.75 (over stoichiometric composition), with an N_2_ partial pressure of 9.5 × 10^−3^ Pa. The TiN1 sample is characterized by the lowest N/Ti ratio. Comparing it with its analog, deposited at a higher temperature (TiN2), a clear difference is observed [25]. As reported by I. Petrov et al. in [26], during reactive bias sputter deposition of titanium nitride, the nitrogen incorporation probability is the result of a competition between different phenomena: on the one side, nitrogen increases due to chemisorption of atomic N generated in the plasma, dissociative chemisorption of N_2_, direct implantation, and recoil implantation; on the other side, a loss of N by preferential resputtering occurs. Thus, since the applied bias voltage does not change, the growth in the N/Ti ratio with increasing temperature can be attributed to the enhanced chemisorption of atomic N generated in the plasma and the dissociative chemisorption of N_2_.

**Figure 2:**
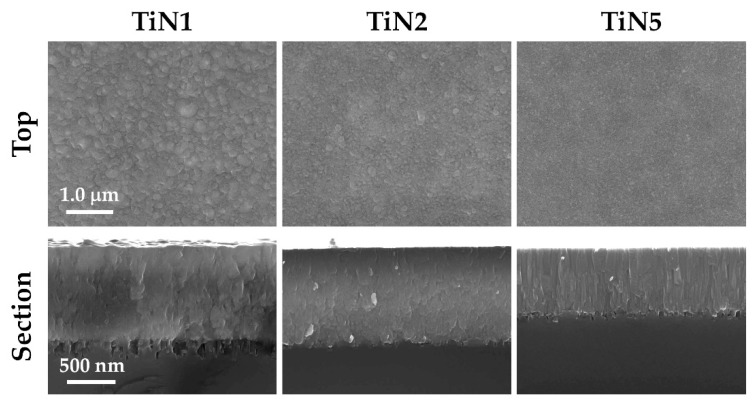
SEM top views and sections of samples TiN1, TiN2, and TiN5 as the deposition temperature and the percentage of reactive process gas vary.

Figure 2 shows the SEM images of the surfaces of TiN1, TiN2, and TiN5 coatings. When comparing TiN1 and TiN2, it is possible to observe a grainy, domed morphology that becomes less evident as the temperature increases: the latter, in fact, favors the surface mobility of the adatoms, making the film denser and smoother [27]. As the N_2_ flow increases (TiN2 and TiN5), the formation of compact TiN films is more probable: TiN5 surface looks relatively flat, and the columns change in both shape and size [28]. This difference is correlated to an evident microstructural variation (i.e., crystalline phases detected) as it will be fully described below. Therefore, substrate temperature and the concentration of N_2_ and Ar during sputtering ought to be responsible for the diverse surface morphologies of the TiN coatings obtained.

Selected experimental conditions were applied to deposit TiN_x_ coatings on alumina porous substrates. Unlike what was observed for silicon supports, in this case, the delamination of the films was observed, and consequently, it was not possible to perform any characterizations. The main criticality was found in the coupling of materials that respond very differently to thermal and mechanical solicitations. In fact, great attention is required since deformations and stress can be generated during both the deposition step and the operational life of the prototype, given the thermal cycles to which it should be subjected [29]. The stresses, in turn, can be detrimental for film/substrate adhesion, which is an essential requirement since delamination, even if only partial, would make the membrane completely ineffective.

The intrinsic adhesion of a film depends on the chemistry of the substrate, which affects the nature of the bonds (i.e., covalent, metallic, ionic) to be established with the growing film. Then, it is influenced by different stress states in the coating/substrate system: internal stress, which is due to the deposition process, the coating microstructure, its thickness, mechanical properties, etc.; external stress that mostly results from structural misfit, plastic or creeps deformation, chemical reactions, phase transformation, precipitation, etc.; and thermal stress, due to the difference in the thermal expansion coefficients of film and substrate and the deposition at high temperature [30].

An important component of the internal stress originates from the energetic bombardment of the growing layer by atomic/molecular species and ions and by energetic non-depositing particles. Indeed, it causes lattice distortion, and thereby it generates compressive stress. Even if the produced HiPIMS fcc TiN film texture is mostly (111), thus confirming a moderate ion bombardment [31], the overall stress level of the samples produced here is probably higher than that of the samples presented in the literature by C. Kura et al. [14].

Given this relatively high level of the overall residual stress, it should be necessary to understand why, when switching from Si to Al_2_O_3_, film/substrate adhesion failed. This could be partially attributed to thermal stress. Indeed, the thermal stress component derives from the high deposition temperatures (here 300 °C) and entails the development of tensile or compressive stress during sample cooling down to ambient temperature. It depends on the thermal expansion coefficients of both the coating (α_f_) and substrate (α_s_). Whether the average value of α_f_ is higher than α_s_, tensile stress is produced after cooling down, as is the case for TiN (α_f_ = 7.2 × 10^−6^ K^−1^) [31] on Si (α_s_ = 2.6 × 10^−6^ K^−1^) [31]. On the contrary, if the average value of α_f_ is lower than α_s_, compressive stress is generated after decreasing temperature, as is the case for TiN on -Al_2_O_3_ (α_s_ = 12.662 × 10^−6^ K^−1^) [32]. This could, to some extent, explain the different adhesion behaviors when moving from silicon to alumina substrates.

**Figure 3:**
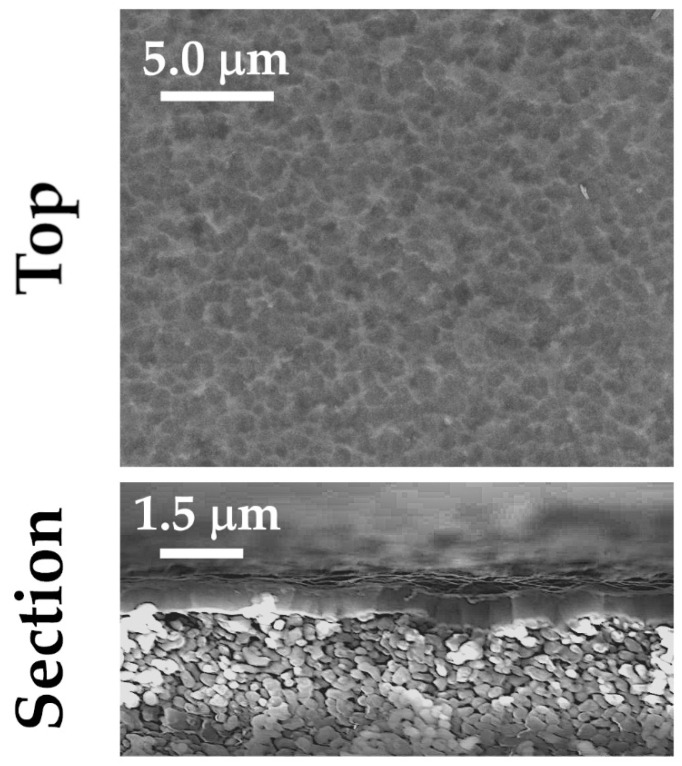
SEM micrographs of samples TiN4A deposited on alumina.

In Figure 3, SEM micrographs of as-deposited TIN4A coating on porous alumina substrate are shown. The same working parameters used for growing TiN4 film were selected, except for the deposition duration that was reduced from 120 to 65 min in order to grow a thinner coating (Table 1). In this case, a suitable film/substrate adhesion was observed: this behavior could be due to the thickness reduction. Indeed, since the thermal expansion coefficient of the film depends on the thickness [30,33], it is reasonable to believe there is a contribution of the thermal stress component change on TiN4A film’s adequate adhesion to the alumina substrate.

In this context, an in-depth adhesion study on the considered film/substrate systems would certainly be necessary, but this is beyond the scope of this work.

From the surface morphology and section view (Figure 3), the film is columnar, dense, and homogeneous. As reported in Table 2, TiN4A and TiN4 sample compositions are similar. Only a slight reduction in the N/Ti ratio is observed: this behavior could be due to a slightly lower deposition temperature since alumina substrate is a thicker and better thermal insulator than silicon, and it was just resting on the sample holder heating surface.

**Figure 4:**
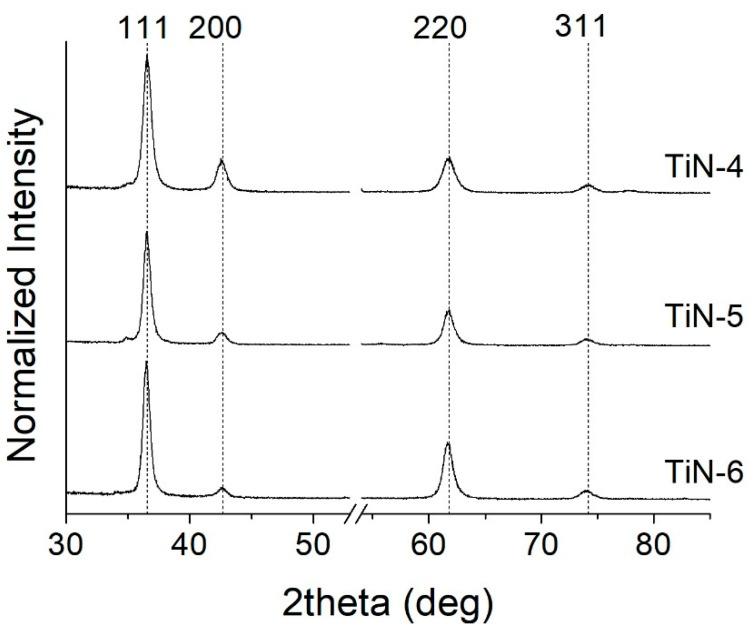
XRD spectra deposited with N_2_ > 1.2 sccm.

TiN_x_ samples deposited on Si (100) wafer substrates under different N_2_ flow conditions were characterized by X-ray diffraction. Analyses indicate that samples deposited with a nitrogen flux >1.2 sccm (TiN4, TiN5, and TiN6) have a face-centered cubic phase (ICSD #604220 TiN, Fm-3m space group, a = 4.24 Å [34]) as depicted in Figure 4 and Table 3. Reflections (111), (200), (202) and (311) are clearly distinguishable at 2θ values 36.7°, 42.6°, 61.8°, and 74.2° respectively. The abscissa axis was interrupted near the peak of the silicon substrate to prevent confusion.

As reported in Table 3, films obtained under high N_2_ flow show a preferential orientation in the crystallographic direction (111). While the reticular parameter remains almost constant, the grain size increases slightly together with the nitrogen flow. Looking at the ratio I_111/220_ for samples TiN4, 5, and 6, the preferential orientation progressively moves towards direction 220. Considering the relationship between preferential orientation and hydride ions diffusion within the Ti-N structure, C. Kura et al. [14] observed that diffusion along the (111) direction is favored over that along the (100) one. In fact, close-packed Ti atoms are characterized by quite a low potential at empty sites due to both short Ti–Ti distances and the lack of N anions. Thus, the predominant (111) preferential orientation obtained in samples TiN4, TiN5, and TiN6 produced herein could improve performance in terms of hydrogen permeability. Therefore, the choice of HiPIMS parameters and deposition temperature was made in order to obtain the mentioned fcc phase (B1) with the (111) preferential orientation and a crystallite size similar to what was reported by C. Kura et al. in [14]. Indeed, upon increasing pulse power and peak discharge current (i.e., by growing the flux of energetic ions), the preferred crystallographic orientation in metal nitrides that exhibit the B1 structure changes from (111) to (100). This is consistent with the conception that a more intense energetic bombardment enhances surface diffusion allowing for the adatoms to accommodate in the (100) planes, which display a lower surface free energy than that of the (111) planes [35,36].

**Figure 5:**
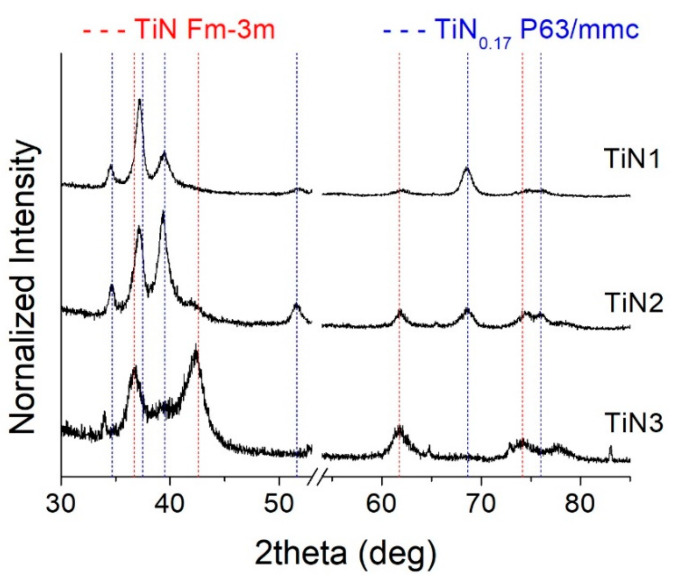
XRD patterns collected from samples TiN1, TiN2, and TiN3, deposited at low nitrogen flow.

On the other hand, low N_2_ flux samples (TiN1, TiN2, and TiN3) show a different behavior, as visible in Figure 5. In particular, TiN3 (N_2_ = 1.2 sccm) represents a sort of transition sample between two different microstructural equilibria. Indeed, it still shows a predominantly face-centered cubic structure but exhibits a different preferential orientation, i.e., (200), which is typical for the corresponding bulk material used as reference. Then, weak signals coming from a second phase, rich in Ti, have a hexagonal symmetry and space group P63/mmc [37] (ICSD #644765).

Instead, TiN2, which was deposited with a nitrogen flow of 1 sccm, shows a biphasic, cubic, and Ti-rich hexagonal crystalline structure. Finally, TiN1 (N_2_ = 1 sccm, deposition T = 140 °C) appears to consist almost entirely of hexagonal phase, missing the signals clearly belonging to the cubic structure. The hexagonal phase is similar to the α-Ti one, with a difference in the presence of nitrogen diffused within the reticule. The solubility of nitrogen in α-Ti was found to be 17 %, and the solid solutions contain randomly distributed nitrogen atoms [37]. Moreover, SEM-EDS analyses, reported in Table 2, confirm the lower amount of N within those coatings deposited at low nitrogen flows.

Therefore, it can be concluded that at higher nitrogen flows, the preponderant and stable phase that forms in the film turns out to be a face-centered cubic structure. Below the threshold value of 1.2 sccm, the structure evolves towards a hexagonal symmetry, rich in titanium and containing a small amount of nitrogen, which becomes predominant at 1 sccm, especially at a low deposition temperature. This is due to the insufficient N_2_ partial pressure during the sputtering process, which is not enough to drive the final structure towards the formation of the cubic nitride phase. Moreover, at a low temperature, the adatoms’ mobility is hindered, thus leading to the formation of a pure α-Ti like structure.

Concerning the prediction of permeability characteristics, it has been reported [14] that the flux of hydrogen through titanium nitride films overcomes that observed for Pd membranes at ambient temperature. Moreover, the hydrogen permeation flux of stoichiometric TiN is smaller than that of slightly N-deficient samples (x ~ 0.7) at T < 100 °C. From the literature, TiN-based membranes are characterized by a pure cubic Fm-3m crystallographic phase with a (111) preferential orientation, and hydrogen permeability is remarkably enhanced by decreasing the grain size down to about 15 nm [12,14]. From Table 2 and Table 3, samples TiN1, TiN2, and TiN3 are sub stoichiometric and have small grains but they contain the Ti-rich hexagonal crystalline structure too.

In light of these concerns, the TiN4 sample was selected to be deposited on alumina to produce a test sample (TiN4A). Indeed, it is approximately stoichiometric, cubic with a (111) preferential orientation, and it shows a relatively small crystallite size. It was used to verify composition and microstructure as they are not necessarily maintained using such a different substrate [29]. As already mentioned, the N/Ti ratio is slightly lower than the TiN4 one.

**Figure 6:**
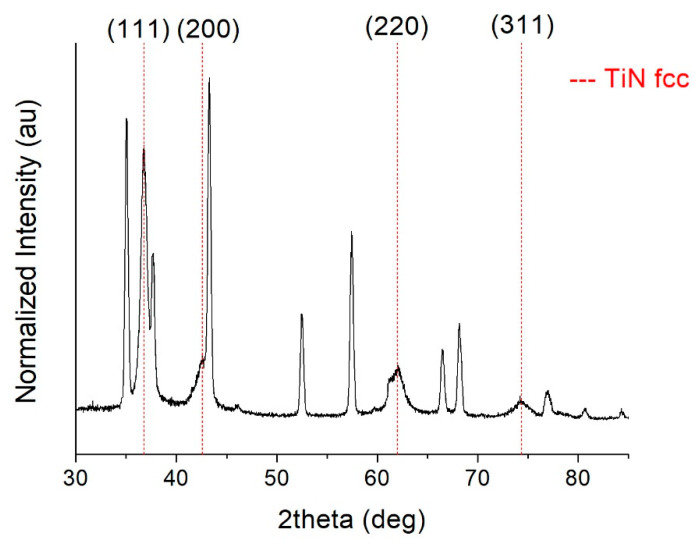
TiN4A XRD spectrum acquired using the grazing angle configuration.

Sample TiN4A exhibited a very predominant (111) preferential orientation, with (111) reflection completely flattening other signals in Brag Brentano mode. Therefore, since the underlying alumina substrate was strongly revealed, the grazing incidence mode was needed to correctly identify the nitride phase, also considering the meager thickness of the coating (Figure 6).

From the analysis of the spectrum, the presence of the stoichiometric well-crystallized fcc Fm-3m TiN phase was highlighted. Peaks were identified and indexed while the remaining signals are attributable to the substrate, consisting of aluminum oxide with a trigonal structure typical of corundum. It also appears well-crystallized. There are no other peaks belonging to unknown and unidentifiable phases. The lattice parameter a = 4.231 Å was estimated, and the resulting crystallite size was about 25 nm. Moreover, in this case, the preferential orientation is in favor of an alleged greater efficiency of hydrogen permeability.

**Figure 7:**
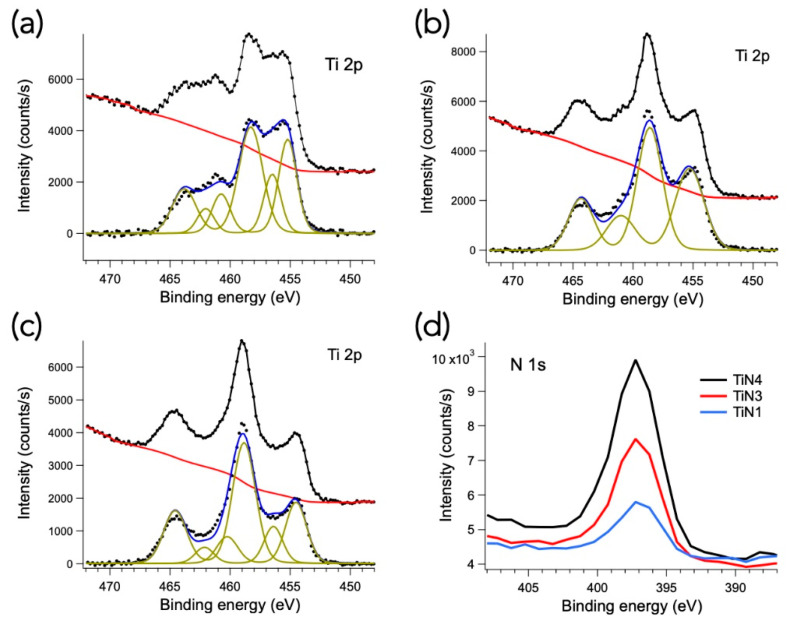
Ti 2p XPS spectra of TiN4 (**a**), TiN3 (**b**) and TiN1 (**c**) samples. The spectra are shown before and after a Shirley-type background (red curves) subtraction. The spectra are also shown decomposed into Voigt-doublets (green), according to the spin-orbit splitting of Ti 2p levels. Panel (**d**): N 1s spectra of the three samples (without background subtraction).

Photoemission spectroscopy measurements were performed on selected TiN_x_ samples. In particular, samples TiN1, 3, and 4 were chosen as they are characterized by different compositions and microstructures. In fact, the TiN4 film is face-centered cubic (Fm-3m), TiN3 shows a mixture of cubic and hexagonal phases, while the TiN1 sample is characterized by a predominant Ti-rich hexagonal phase (P63/mmc). The photoemission spectra from the Ti 2p core levels of the three representatives are shown in Figure 7, both before and after a Shirley-background subtraction. The different contributions of titanium in the system are shown in the background-subtracted spectra, represented by different Voigt-type doublets due to the spin-orbit interaction. In particular, the doublet with the highest bond energy, at about 458 eV, is attributable to oxidized titanium, the intermediate structure, at about 456.5 eV, to Ti-O-N bonds and the doublet with the lowest bond energy, at about 455 eV, is associated with Ti-N [38]. Given the sampling depth of the technique, equal to a few nm, the percentage of oxidized titanium on the surface appears to prevail, as can be expected for ex-situ samples. On the other hand, there is a progressive increase in the Ti-N component with the N/Ti ratio in the samples (i.e., > reactive N_2_ flow). This is also clearly visible from the relative growth of the intensities of N 1s peaks in the three samples, as shown in Figure 7d. We can, therefore, conclude that the photoemission spectroscopy measurements are in good agreement with the SEM-EDS measurements shown in Table 2.

Additionally, especially sub stoichiometric TiN_x_ membranes may be susceptible to oxidation and require modification to their surface properties to eventually display high hydrogen flux. The addition of a precious metal protective film (i.e., Pd or Pd-alloy) to form a multilayer Pd-TiN_x_ architecture on alumina could be extremely useful. Indeed, such a layer might improve both chemical stability and the catalytic activity of the membrane (for H_2_ dissociation and absorption). Moreover, as suggested in [39], a further film between the nitride and the alumina substrate (Pd-TiN_x_-Pd) should be recommended to increase hydrogen re-association and desorption. These very thin films could be easily grown via magnetron sputtering immediately before and after the active layer deposition without it being exposed to air.

### 3.2. Stability under H_2_-Containing Atmosphere

**Figure 8:**
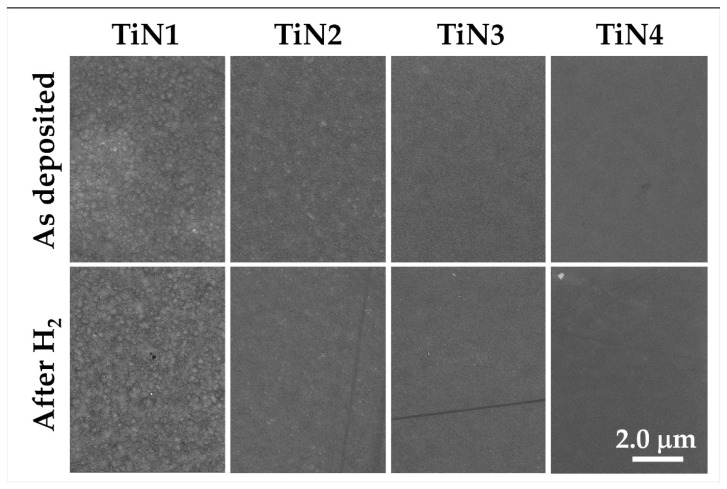
SEM micrographs of samples TiN1, TiN2, TiN3, TiN4 after thermal treatment at 500 °C under H_2_-containig atmosphere for 20 h.

To investigate the chemical stability and robustness under operational conditions typical for hydrogen separation membranes, four representative samples (i.e., TiN1, TiN2, TiN3, and TiN4) were treated at 500 °C for 20 h under an H_2_-containing atmosphere. SEM images of the surface of the treated samples are shown in Figure 8. Apparently, no evident modifications of the surface morphologies could be observed. The film compositions measured by SEM-EDS are reported in Table 2. The stoichiometry of the samples is substantially unchanged with respect to as-prepared coatings.

**Figure 9:**
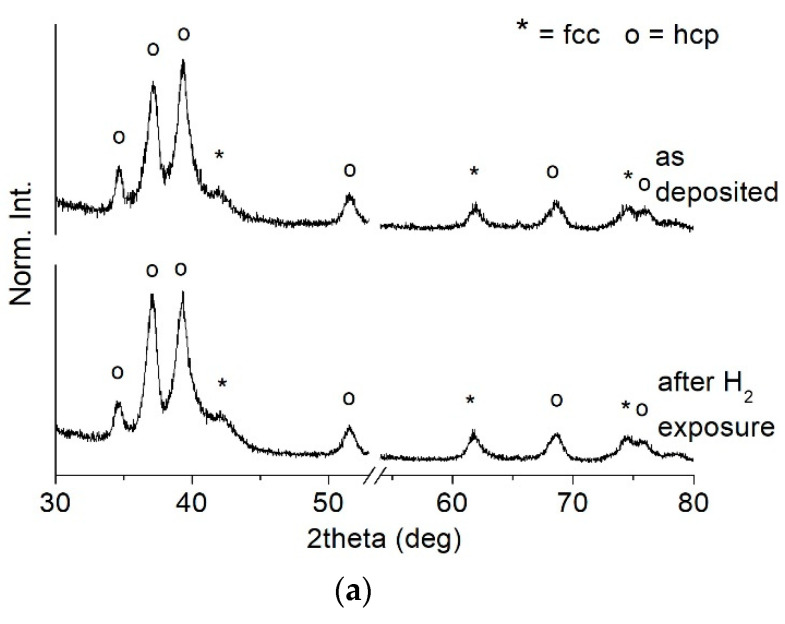

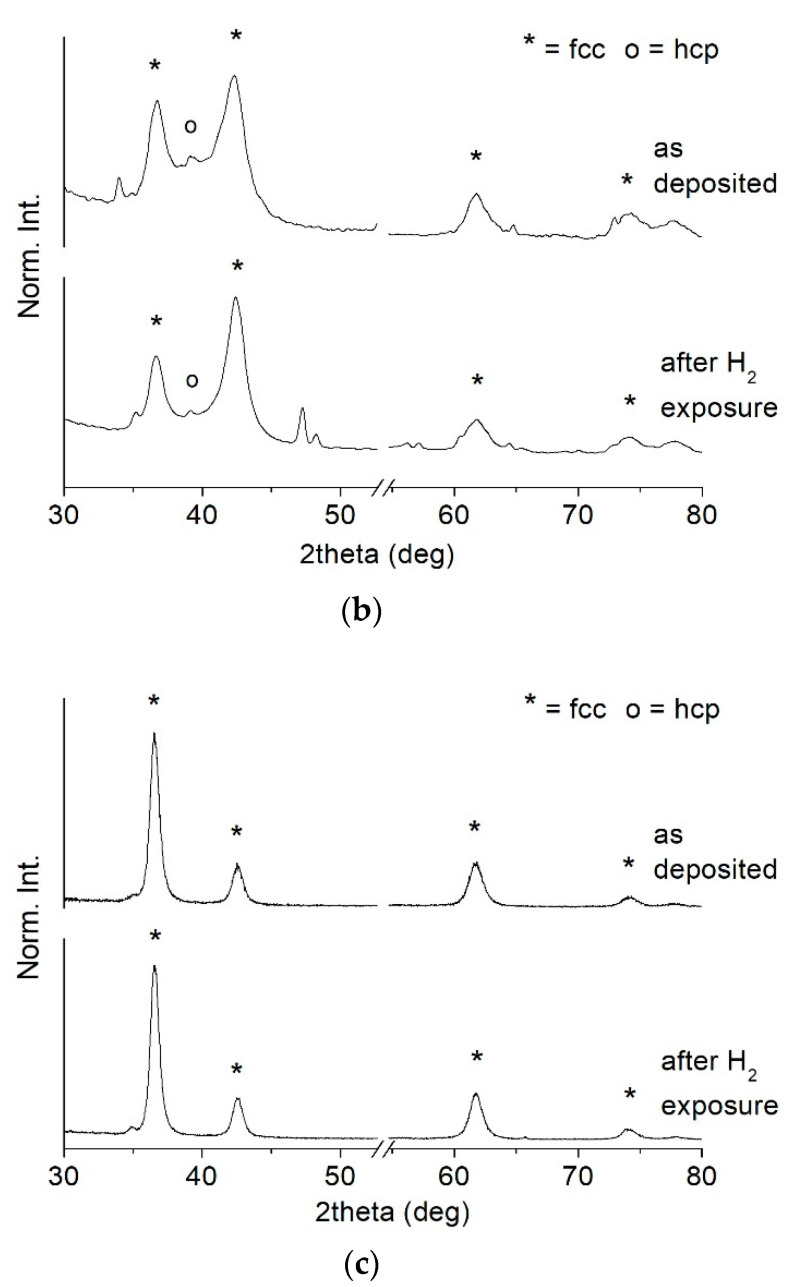
XRD spectra of samples (**a**) TiN2, (**b**) TiN3 and (**c**) TiN4 before and after thermal treatment at 500 °C under H_2_-containig atmosphere for 20 h.

Moreover, TiN_x_ coatings show good microstructural stability, as no significant variations were highlighted for all treated samples, as shown in Figure 9, where XRD profiles of TiN2, TiN3, and TiN4 samples are reported.

There are also no significant variations in the texture, which could be linked to a microstructural rearrangement, nor are there modifications in the position of the high-angle peaks, which would represent a sign of residual stress relaxation [29].

It can be concluded that after the thermal treatment under hydrogen reducing conditions, the microstructure was maintained by all the samples, thus demonstrating suitable chemical robustness for the application of these materials as hydrogen separation membranes.

## 4. Conclusions

This preliminary work represents a valuable starting point to define the guidelines for depositing titanium nitride films via RHiPIMS, given their eventual use as hydrogen separation membranes. Indeed, recent literature papers demonstrated that in suitable conditions, nitride-based thin films are permeable to hydrogen and can selectively separate it. Therefore, this investigation aimed to verify the possibility of growing reliable titanium nitride coatings using the Reactive High Power Impulse Magnetron Sputtering technology (RHiPIMS). As described, a series of TiN_x_/Si samples were deposited and characterized while changing substrate temperature and the reactive gas percentage (N_2_%) during the process. Moreover, a ~500 nm thick, dense TiN coating was deposited on a porous alumina substrate and widely investigated.

HiPIMS is an ionized PVD technology since ions constitute a high percentage of the total flux of the film-forming species. With respect to traditional magnetron sputtering, the elevated ion bombardment affects the coating microstructure thanks to the enhanced adatom mobility. Indeed, it leads to a decrease in the nucleation density, densification, surface smoothening, and changes in the film crystallographic orientation, even when the deposition is performed at low temperature, on electrically grounded coupons and insulating substrates, like alumina.

The main results gained can be briefly summarized as follows.

- With the same process parameters, the substrate temperature change from 140 °C to 300 °C promotes a N/Ti ratio increase (enhanced chemisorption of atomic N plus dissociative chemisorption of N_2_) and a reduction in thickness (i.e., >density).
- While the process N_2_/Ar ratio increases, TiN_x_ film stoichiometry increases (SEM-EDS), but deposition rate decreases (Calotest).
- At high nitrogen flows (>1.2 sccm), the preponderant and stable phase that forms in the film is a face-centered cubic structure (ICSD #604220 TiN, Fm-3m space group) with a (111) preferential orientation. On the other hand, at low nitrogen flows (≤1.2 sccm), the structure evolves towards a hexagonal symmetry and space group P63/mmc (ICSD #644765). This phase is rich in Ti and contains a small amount of nitrogen. It becomes predominant at 1 sccm.
- The sample grown on alumina (TiN4A) is quasi-stoichiometric, and the XRD spectrum shows the presence of a well-crystallized fcc Fm-3m TiN phase. Moreover, a very predominant (111) preferential orientation is exhibited.
- XPS analyses confirmed that TiN_x_ membranes might be susceptible to oxidation that could hinder hydrogen permeation. The addition of protective films (i.e., Pd or Pd-alloy) to form a multilayer Pd-TiN_x_-Pd architecture on alumina could be extremely useful.
- Chemical robustness was confirmed after the thermal treatment at 500 °C for 20 h under a hydrogen-containing atmosphere: composition and microstructure were maintained by all the samples, thus highlighting their stability under reducing conditions.

## Figures and Tables

**Table 1:** Thickness and deposition rate of grown titanium nitride samples while varying the quantity of nitrogen during the sputtering process.

Sample	Substate	Dep. T (°C)	Duration (min)	N_2_ Partial p (×10^−3^ Pa)	N_2_/Ar %	Thickness (nm)	Dep. Rate (nm/min)
TiN1	Si	140	120	2.8 (1 sccm)	0.6	1207	10.1
TiN2	Si	300	120	2.8 (1 sccm)	0.6	1160	9.7
TiN3	Si	300	120	3.5 (1.2 sccm)	0.7	1080	9.0
TiN4	Si	300	120	4.4 (1.5 sccm)	0.9	1090	9.1
TiN5	Si	300	120	6.2 (2 sccm)	1.2	643	5.4
TiN6	Si	300	120	9.5 (3 sccm)	1.8	430	3.6
TiN4A	Al_2_O_3_	300	65	4.4 (1.5 sccm)	0.9	590	9.1

**Table 2:** SEM-EDS compositional analyses of TiN_x_ samples deposited on silicon.

Sample	As Deposited	After H_2_
TiN1	0.43	0.44
TiN2	0.66	0.62
TiN3	0.85	0.87
TiN4	1.1	1.09
TiN5	1.25	not treated
TiN6	1.75	not treated
TiN4A	1.01	not treated

**Table 3:** Microstructure, lattice parameters, crystallite size and texture of TiN_1-x_ coatings.

Sample	Structure	Phase%	Lattice Parameters		Grain Size	Texturing	
		%wt	a	c	(nm)	Fm-3m	P63/mmc
			(Å)	(Å)		I_111/220_	I_002/101_
TiN (ref) ICSD #604220	Fm-3m	-	4.24	-	-	1.4	-
Ti_0.83_N_0.17_ (ref) ICSD #644765	P63/mmc	-	2.969	4.777	-	-	0.27
TiN1	P63/mmc	100	2.989	4.835	16	-	2.1
TiN2	Fm-3m	5	4.277	-	-	-	
	P63/mmc	95	2.989	4.833	13	-	0.87
TiN3	Fm-3m	95	4.237	-	12	2.4	-
	P63/mmc	5	-	-	-	-	-
TiN4	Fm-3m		4.24	-	26	3.85	-
TiN5	Fm-3m		4.238	-	30	3.24	-
TiN6	Fm-3m		4.238	-	34	2.5	-

## Data Availability

Data are available on request due to privacy restrictions. The data presented in this study are available on request from the corresponding author.
